# Electrospun Nanofiber-Scaffold-Loaded Levocetirizine Dihydrochloride Cerosomes for Combined Management of Atopic Dermatitis and Methicillin-Resistant *Staphylococcus Aureus* (MRSA) Skin Infection: In Vitro and In Vivo Studies

**DOI:** 10.3390/ph18050633

**Published:** 2025-04-27

**Authors:** Rofida Albash, Samer Khalid Ali, Rehab Abdelmonem, Ahmed M. Agiba, Renad Aldhahri, Asmaa Saleh, Amira B. Kassem, Menna M. Abdellatif

**Affiliations:** 1 Department of Pharmaceutics, College of Pharmaceutical Sciences and Drug Manufacturing, Misr University for Science and Technology, Giza 12585, Egypt; 2 College of Pharmacy, Al-Ayen Iraqi University, Nasiriyah 64001, Iraq; samer.ali@alayen.edu.iq; 3 Department of Industrial Pharmacy, College of Pharmaceutical Sciences and Drug Manufacturing, Misr University for Science and Technology, Giza 12566, Egypt; rehab.abdelmonem@must.edu.eg (R.A.); menna.abdallatif@must.edu.eg (M.M.A.); 4 School of Engineering and Sciences, Tecnologico de Monterrey, Monterrey 64849, Mexico; ahmed.agiba@tec.mx; 5 Department of Pharmaceutical Care, King Abdullah bin Abdulaziz University Hospital, Riyadh 13412, Saudi Arabia; raaldhahri@kaauh.edu.sa; 6 Department of Pharmaceutical Sciences, College of Pharmacy, Princess Nourah bint Abdulrahman University, P.O. Box 84428, Riyadh 11671, Saudi Arabia; asali@pnu.edu.sa; 7 Clinical Pharmacy and Pharmacy Practice Department, Faculty of Pharmacy, Damanhour University, Damanhour 22514, Egypt; amirabisher@gmail.com

**Keywords:** atopic dermatitis, MRSA skin infection, atomic force microscopy, cerosomes, confocal laser scanning microscopy, levocetirizine dihydrochloride, nanofiber

## Abstract

**Objectives:** In this study, we aimed to incorporate levocetirizine dihydrochloride (LVC) into electrospun nanovesicle-in-nanofiber (NF) scaffolds for combined management of atopic dermatitis and methicillin-resistant *Staphylococcus Aureus* skin infection, to sustain LVC release for continuous skin improvement. **Methods:** Firstly, LVC was encapsulated in cerosomes (CERs) by employing a thin-film hydration approach using a 2^1^.3^1^ factorial design. CERs were assessed by calculating entrapment efficiency (EE%), particle size (PS) and polydispersity index (PDI). In addition, the optimized CERs were further subjected to stability evaluation. After that, the optimized CERs were incorporated into polyurethane nanofibers (NFs) using a coaxial electrospinning technique. An in vitro release assay was used to calculate the amount of LVC released from the LVC-NFs and the optimized CERs-NFs. For morphological assessment of NFs, LVC-NFs and CERs-NFs were subjected to transmission electron microscopy, scanning electron microscopy, and confocal laser scanning microscopy. Atomic force microscopy was utilized to evaluate the roughness of CERs and both NFs. The optimum formulation was further subjected to in vivo study. **Results:** The optimum CERs exhibited an EE% of 65.03 ± 1.07%, a PS of 680.00 ± 39.50 nm, and a PDI of 0.51 ± 0.04. LVC was released in a sustained manner from CERs NFs. Further, a dermatokinetic study confirmed that CERs-NFs sustained the infiltration of LVC, compared with the other groups. Finally, a safety assessment showed that all formulations were safe when topically applied to rat skin. **Conclusions:** In conclusion, AD and MRSA skin infections may be cured by employing electrospun nanofiber-scaffold-loaded LVC CERs, which can thus be regarded as a promising system.

## 1. Introduction

Atopic dermatitis (AD) is a pruritic, inflammatory condition that can range from mild to severe. Patients usually go through periods of remission interrupted by flares, which are acute inflammatory relapses. A family history of allergic illnesses has been shown to be a good predictor of the onset of AD, which is the most common reason for such atopic disease [1]. The prevalence of AD differs with age, sex, and region, with children demonstrating higher rates compared to adults. Globally, females are slightly more affected than males. Moreover, AD patients are frequently colonized by *Staphylococcus Aureus*, including methicillin-resistant *Staphylococcus Aureus* (MRSA), which exacerbates disease severity and poses significant treatment challenges [1]. Studies show that MRSA colonization rates in AD patients are significantly higher than in healthy populations, ranging from 4% to 18%, depending on geographical and demographic factors. Pediatric AD patients particularly exhibit elevated MRSA colonization rates, predisposing them to invasive infections and systemic complications [2]. Moreover, MRSA produces virulence elements that increase skin barrier dysfunction, which is one way that MRSA contributes to AD [2]. It has previously been established that a lack of typical ceramides in the stratum corneum (SC) is a key etiologic reason for the dry, barrier-damaged skin seen in AD patients [3].

Further, it has been shown that sphingoid, a fundamental constituent of ceramides, may have a defensive effect against Gram-positive bacteria like *Staphylococcus Aureus*, preventing them from colonizing and infecting the skin [4]. Ceramides are the least polar and most hydrophobic lipids in the SC, giving it its barrier qualities [5]. Cerosomes (CERs) are ceramide-containing nanovesicles that have previously been reported as a successful drug-delivery nanosystem for the curing of psoriasis, topical fungal infections, alopecia, MRSA skin infection, and hirsutism [6,7,8,9,10,11].

Levocetirizine dihydrochloride (LVC) is an antihistaminic medication that is commonly used to manage allergic rhinitis, hay fever, and idiopathic urticaria. It has a higher attraction for H_1_ receptors than its enantiomer cetirizine. In vitro studies of LVC reveal that it modifies the inflammatory mediators produced by eosinophils [12]. In a previous study, LVC was fabricated into vesicles for its action against AD, to decrease LVC side effects such as dry mouth, drowsiness, and tiredness [12]. The topical route also provides an effective strategy to overcome LVC’s intolerably bitter taste [13]. It is worth noting that therapy for AD disease necessitates the production of a topical drug delivery system that maintains skin improvement over time [14]. Further, recent publications by the authors have highlighted the activity of LVC against MRSA skin infection when used topically [15].

Electrospun nanofibers (NFs) are gaining popularity due to their unique features. Natural and synthetic polymers, as well as their combinations, can be used to make NFs. NFs exhibit a high surface area, which allows hydrophilic or lipophilic drugs to be delivered efficiently. Various characteristics such as polymer concentration, polymer type, morphology, fiber diameter, surface roughness, and porosity can be changed to alter drug release profiles. The selection of polymer(s) is a critical factor in achieving the required drug release qualities [16]. Several researchers have successfully demonstrated the desired sustained effect of NFs for the treatment of topical candidiasis using sertaconazole, application of the wound-healing property of phenytoin, and the effective eradication of *Staphylococcus Aureus* by moxifloxacin hydrochloride [17,18,19].

Polyurethane (PU) has been widely employed in the manufacture of NF scaffolds. PU’s extremely variable chemistry enables the creation of materials with precisely regulated mechanical, physicochemical, and biodegradation qualities, which can be achieved by electing the right monomers and using hard and soft components. For instance, adding urea linkages or aromatic groups to chain extenders increases hard-segment contacts by forming bidentate hydrogen bonds between adjacent chains or stacking p-bonds between adjacent aromatic rings. The mutual aspect of all interaction within the hard segment domains establishes the thermal, mechanical, and hydrolytic behavior [20].

To develop drug delivery techniques based on electrospinning, a drug should be mixed with the polymer in the electrospinning mixture. Because of the large surface area of electrospun mats, solvent evaporation is quick and effective; this gives the integrated drug little time to recrystallize, favoring the development of amorphous dispersions. The drug is then diffused into the dissolving liquid by the mat. The drug is consistently dispersed through the polymer matrix in a matrix diffusion-control system. The infiltration of the drug via the matrix is controlled by the distribution of the dissolution media throughout the matrix phase. Further, the system parameters and the thickness of the membranes might additionally have an impact on the rate of drug release [21].

Coaxial electrospinning is a modification of the conventional electrospinning process [22]. It is a new type of electrospinning that employs two concentrically supported capillaries to ensure the creation of core-shell fibers. The primary rationale for using coaxial-electrospinning in controlled release is to avoid the drawbacks of single-nozzle electrospinning in encapsulating weak, water-soluble bioactive compounds that are important in regenerative medicine. Other benefits of coaxial electrospinning include a more sustained effect of the encapsulated medications as well as one-step co-encapsulation of several pharmaceuticals with differing solubility aspects [23].

As far as we know, the effect of coaxially produced electrospun nanovesicle-in-NF-scaffold-loaded LVC upon its combined management of AD and MRSA skin infection has yet to be discussed in any scientific paper. Consequently, LVC, which is a hydrophilic drug, was fabricated into nanovesicles (CERs) and then incorporated into hydrophobic PU electrospun NFs to sustain its effect. CERs were optimized using factorial design 2^1^.3^1^, in which the categoric factor ceramide type (X_1_) with two levels (IIIB and IV) and the numeric factor phospholipid (PC) amount (X_2_) with three levels (50, 75 and 100 mg) were selected as independent variables, and EE% (Y_1_), PS (Y_2_) and PDI (Y_3_) were chosen as dependent variables. The optimized CERs were examined in further investigations and then incorporated into NFs. The fabricated NFs were studied in terms of fiber size and surface morphology. The NF-loaded CERs were further evaluated via an in vitro release study and an in vivo study. A histopathological assessment was accomplished to assess the safety of NF-loaded CERs.

## 2. Results and Discussion

### 2.1. Analysis of 2^1^.3^1^ Factorial-Design

The formulae of CERs were optimized utilizing 2^1^.3^1^ factorial design, applying Design-expert^®^ version 13 (Stat Ease, Inc., Minneapolis, MN, USA), as demonstrated in Table 1. The model’s potential for navigating the design space was assured with adequate precision. The adequate precision for all dependent variables was seen to be a value greater than four. All dependent variables’ predicted *R*^2^ values and adjusted *R*^2^ values agreed well.

### 2.2. EE%

**Figure 1:**
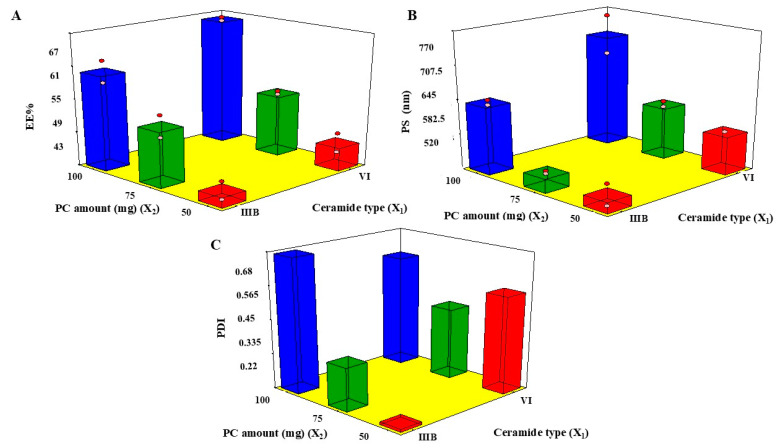
Three-dimensional plots of the effects of ceramide type (X_1_) and PC amount (X_2_) of LVC-CERs. Three-dimensional plots of the effects of ceramide type (X1) and PC amount (X2) on (**A**) EE%, (**B**) PS, and (**C**) PDI of LVC-CERs.

The influence of X_1_ and X_2_, on the EE% of LVC in CERs is displayed in Table 2 and Figure 1A. The EE% of LVC within CER formulations ranged from 43.15 ± 3.23% to 65.03 ± 1.07%, as indicated in Table 2. Further, the drug loading percentage ranged from 11.81 ± 0.67% to 15.53 ± 0.98%. The results demonstrated that ceramide type (X_1_) exerted a substantial impact (*p* < 0.0001) on the EE% of CERs, in which the highest EE% was found with CERs fabricated with ceramide VI, followed by those fabricated with ceramide IIIB. Ceramide VI is composed of a saturated phytosphingosine chain acylated with a long alpha-hydroxy stearic acid [5]. Further, ceramide IIIB is constituted of a phytosphingosine acylated with oleic acid; moreover, it possesses an unsaturated bond in its fatty acid chain [24]. Therefore, this unsaturation in ceramide IIIB might affect its rotation, creating leakier CERs as the packing of the CERs might not be tight [25].

Regarding PC amount (X_2_), it was found that EE% was boosted by augmenting the PC amount from 50 mg to 100 mg, as an elevated system viscosity might be predictable at a higher PC amount which may retard the diffusion of LVC [26]. These outcomes correlated with those of Albash et al., who stated that EE% increased as PC increased, a finding which could be ascribed to the increase in the distance presented for LVC encapsulation into CERs [27].

### 2.3. PS

The z average was assessed, as shown in in Table 2 and Figure 1B [28]. The PS of CERs ranged from 531.66 ± 19.39nm to 680.00 ± 39.50 nm. It has been previously stated that vesicles < 300 nm have a potential impact on conveying the drug via the skin, as they were capable of carrying their constituents into the skin. In contrast, the fabrication of nano-vesicles (100–200 nm) might allow transfer via the skin anatomical constraints [29]. These previous findings indicated that CERs might be a good carrier for the enhancement of LVC topical delivery.

Regarding ceramide type (X_1_), PS was bigger in CERs containing VI, compared with CERs containing IIIB. These results were in accordance with the EE% outcomes, as higher-PS CERs were found in CERs with higher EE% values [27]. Moreover, the molecular weights of ceramide IIIB and ceramide VI were 582 g/mol and 600 g/mol, respectively. However, there was a small variance in the molecular weight, resulting in the formation of larger CERs in the case of CERs created by ceramide VI. As formerly reported, the increase in the molecular weight augmented the viscosity of the dispersion, possibly developing aggregates and augmenting the PS [30].

With regard to PC amount (X_2_), the PS of CERs increased in line with increases in the PC amount from 50 to 100 mg; hence, the amount of surfactant (Pluronic F127) would not be sufficient to diminish the interfacial tension leading to increased PS [31]. Further, the diminished PS at lower PC amounts could be ascribed to the elevated concentrated energy distribution in dilute dispersion, which accordingly decreased the PS of the CERs [32].

### 2.4. PDI

A population of entirely mono-dispersed particles has a PDI value of zero, whereas a population of highly poly-dispersed particles has a value of unity [25]. Both inspected factors, ceramide type (X_1_) and PC amount (X_2_), revealed significant effects (*p*-values 0.035 and 0.0051, respectively), as illustrated in Figure 1C. PDI values of the measured CERs ranged from 0.21 ± 0.02 to 0.61 ± 0.08 (Table 2). This revealed that CERs were polydisperse, but within proper limits [33]. Considering ceramide type (X_1_) and PC amount (X_2_), it was found that by using ceramide VI and increasing PC amount, the PDI values of CERs increased. These findings correlated with PS measurements because using ceramide VI and a high PC amount increased PS, resulting in a simultaneous increase in PDI values. These findings conformed with results of previous research which found that an increase in PS of the prepared nano-dispersion might be accompanied by an increase in PDI values [34].

### 2.5. Determination of the Selected CERs

Using Design Expert^®^ software version 13, we obtained the desired levels of the variables to generate LVC-CERs with the highest EE% and PS, and the lowest PDI. Therefore, the optimized formula was CER 6, which included 100 mg PC and ceramide VI. Thus, CER 6 was selected for additional studies, such as ZP with a value of −12.00 ± 0.01 mV.

### 2.6. Effect of Storage

Visual examination of the preserved system revealed no evidence of vesicle accumulation or sedimentation during storage. Additionally, PS, PDI, EE%, and ZP values of the stored CER 6 were 689.40 ± 19.50 nm, 0.516 ± 0.03, 64.34 ± 1.85% and −11.3 ± 0.50 mV, respectively, values which deviated only insignificantly from those of the fresh CERs.

### 2.7. In Vitro Release

**Figure 2:**
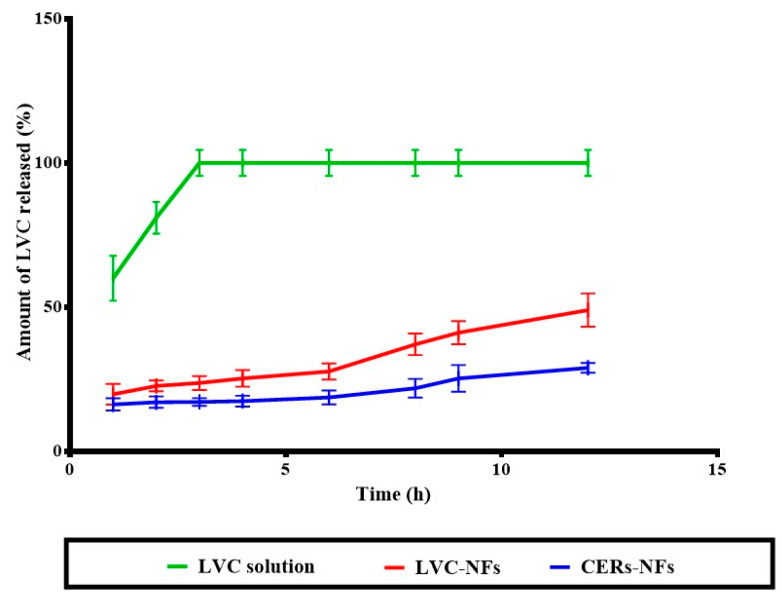
In vitro drug release study.

The primary consideration in both AD and MRSA skin infection management is to develop a sustained single-dosage form for successful treatment. As can be seen in Figure 2, LVC from both NFs showed significant, sustained release, compared with the LVC solution. However, statistical analysis showed that CERs-NFs indicated a significant, sustained drug release profile, compared with LVC-NFs. This could be ascribed to the presence of PC and ceramide, as both constituents might impart a hydrophobic character to NFs and subsequently sustain the release of LVC from NFs [35].

### 2.8. Morphological Assessment

#### 2.8.1. Scanning Electron Microscopy

**Figure 3:**
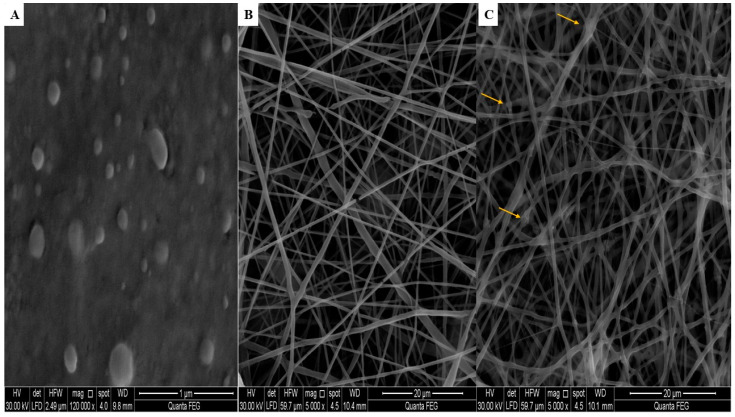
Scanning electron micrographs for (**A**) CERs, (**B**) LVC-NFs, and (**C**) CERs-NFs.

SEM graphs of the CER 6, LVC-NFs, and CERs-NFs were obtained and examined. CERs after lyophilization showed spherical morphology, as illustrated in Figure 3, while both LVC-NFs and CERs-NFs showed elongated smooth and uniform NFs without aggregation, with average diameters of 363.00 ± 49.38 nm and 493.33 ± 22.50 nm, respectively. SEM analysis showed that the incorporation of CERs into the NFs resulted in a significant increase in NF diameter. SEM graphs of CERs-NFs showed some beaded areas (indicated by yellow arrows). This could be ascribed to the greater hydrophobicity of ceramides integrated into CERs, which resulted in augmenting the viscosity of the fabricated NFs. These results were in line with findings previously reported in the literature [36].

#### 2.8.2. Atomic Force Microscopy (AFM)

**Figure 4:**
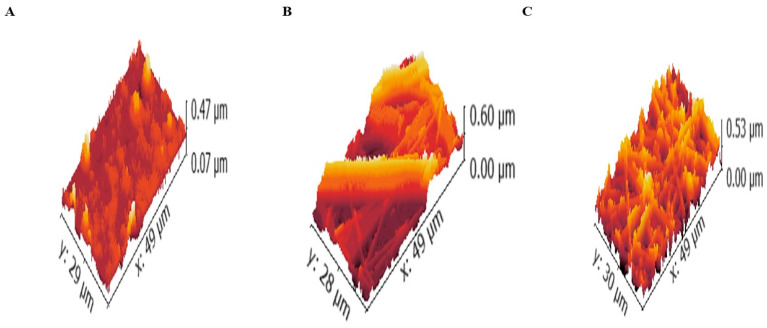
Atomic force micrographs of (**A**) plain fibers (without CERs or LVC), (**B**) LVC-NFs, and (**C**) CERs-NFs.

After SEM analysis, the surfaces of plain fibers (without CERs or LVC), LVC-NFs and CERs-NFs were investigated using Gwyddion^®^ 2.59, open source software, Brno, Czech Republic, http://gwyddion.net/ accessed on 18 November 2023. The micrograph was measured. The resolution of the 3D micrograph module was set to 1400 × 960 pixels for LVC-NFs and CERs-NFs to facilitate comparison between them. The evaluated surface parameters for comparison are depicted in Table 3 and Figure 4. It was found that the incorporation of CERs-loaded LVC led to a decrease in R_a_ to some extent. The value decreased from 46.27 ± 5.40 nm to 42.06 ± 4.00 nm after insertion of CERs-LVC, indicating that LVC in the NFs mat is high and deep and possesses protrusion/hollows [37]. As the value of R_q_ followed R_a_, the values of R_t_ decreased for CERs-NFs rather than LVC-NFs. It was therefore clarified that both R_p_ and R_tm_ followed the trend of R_t_. This difference in trends among R_a_ and both R_t_ and R_p_ is described by their definitions [38].

#### 2.8.3. Transmission Electron Microscopy (TEM)

**Figure 5:**
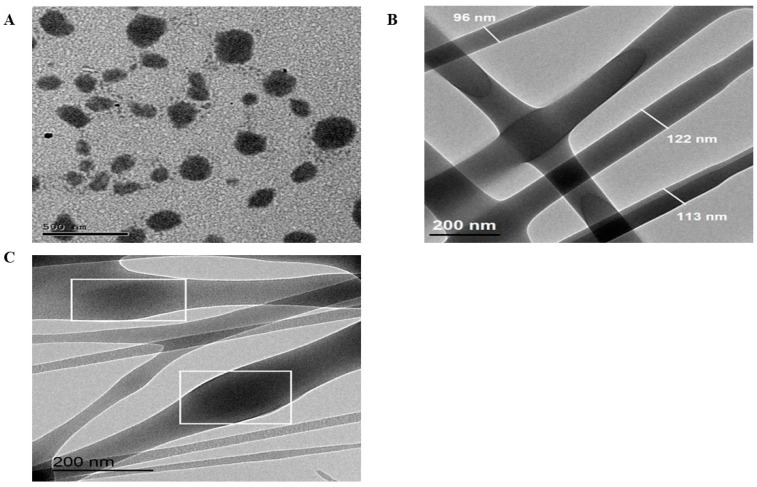
Transmission electron microscope images of (**A**) CERs, (**B**) LVC-NFs and (**C**) CERs-NFs.

Figure 5A displayed CERs with elongated shape. This was also found in a previous study whose authors noticed that the addition of PC to ceramide produced elongation of their prepared vesicles related to the presence of ceramide into the PC, complemented by increase in the surface rigidity [5]. Both NFs showed elongated fiber morphology. Figure 5C shows that CERs as core shells were encapsulated entirely in PUs NFs.

#### 2.8.4. Confocal Laser Scanning Microscopy (CLSM)

**Figure 6:**
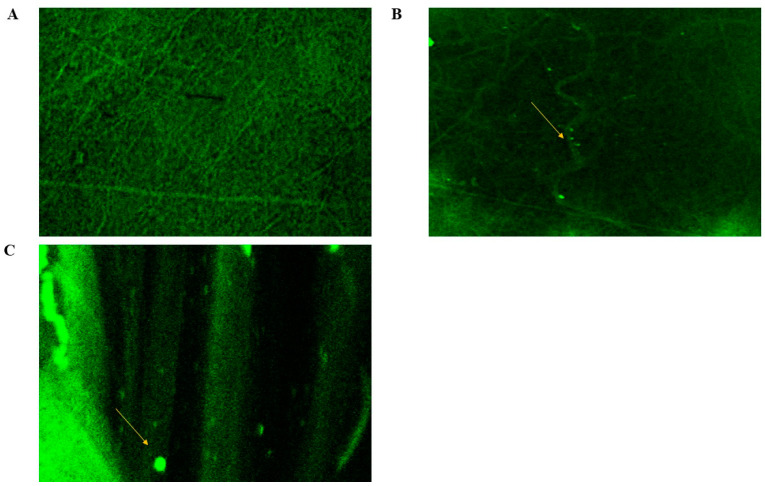
CLSM photographs of sections of stratum corneum handled with FDA-NFs: (**A**) blank NFs; and (**B**,**C**) CERs-loaded NFs.

The shapes of the fabricated CERs-in-NFs was revealed by CLSM scans, as shown in Figure 6B,C, and compared with those of LVC-NFs (Figure 6A). Fluorescein diacetate (FDA) dye was employed as an alternative to LVC to prove the effective integration and homogeneous distribution of CERs in NFs. The distribution of CERs within NFs was in harmony with the SEM, where CERs exhibited excellent distribution with beaded regions (indicated by the yellow arrows (Figure 6B,C)) due to the existence of PC in CERs [18].

### 2.9. In Vivo Studies

#### 2.9.1. Dermatokinetic Study

**Figure 7:**
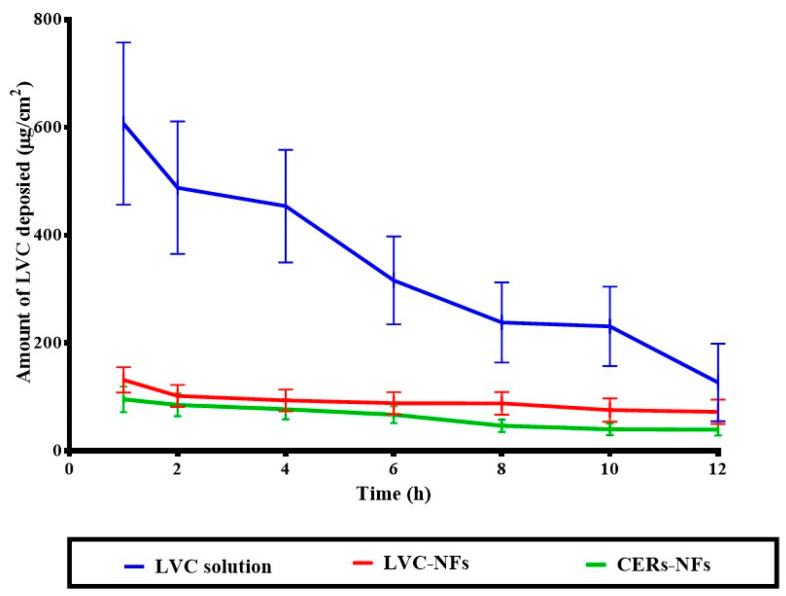
Dermatokinetic study of LVC solution, LVC-NFs, and CERs-NFs.

The results of our dermatokinetic study of LVC from LVC-NFs and CERs-NFs are illustrated in Figure 7. Statistical assessment demonstrated that there was a substantial difference (*p* < 0.05) between the AUC_0–12_ values of LVC-treated groups. The calculated AUC_0–12_ values were 5026.16 ± 301.71 μgh/cm^2^, 2417.96 ± 703.61, and 1970.24 ± 760.84 μgh/cm^2^ for LVC solution, LVC-NFs, and CERs-NFs, respectively. However, there were no significant differences between the T_max_ values of the fabricated systems. The ability of NFs to sustain the deposition of LVC from both LVC-NFs and CERs-NFs was related to the capability of PU to release LVC in a controlled manner from NFs. These results agreed with the findings of Jatoi et al., who confirmed that the addition of PU to NFs sustained the antibacterial effect of silver nanoparticles/zinc oxide (Zn-Ag) composite nanoparticles incorporated into NFs as wound dressing [39]. In addition, we speculate that the presence of ceramide VI might have a synergistic effect on the retention of LVC from CERs-NFs as it is an extremely hydrophobic lipid matrix. Hence, the addition of both PU and ceramide VI successfully aided in controlling the LVC release profile from CERs-NFs and might aid in the enhancement of LVC retention inside CERs-NF. Moreover, it was observed from the outcomes that the amount of LVC deposited was diminished by prolonging the time (12 h). This might be ascribed to the cellular uptake of skin becoming saturated; subsequently, the amount of LVC moved into the blood from the skin exceeded the amount of LVC infiltered through the skin, resulting in a decline in skin deposition [40].

#### 2.9.2. Histopathological Study

**Figure 8:**
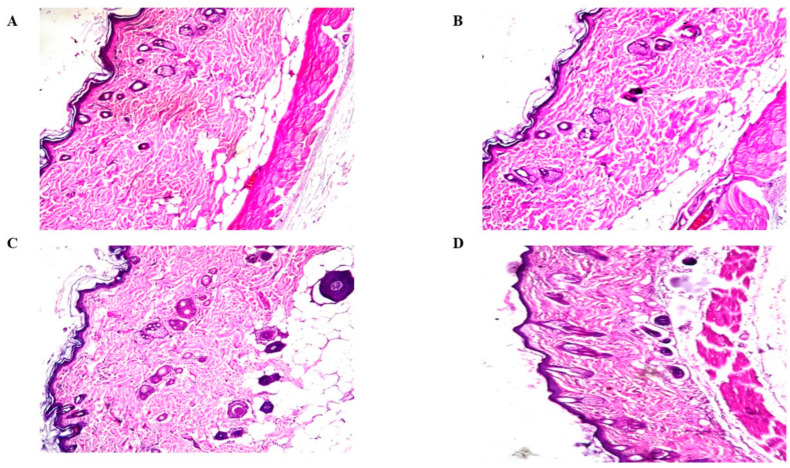
Histopathological sections of control group rat skin (group I) (**A**), LVC solution treated rat skin (group II) (**B**), LVC-NFs treated rat skin (**C**), and CERs-NFs (**D**).

In a microscopy assessment of groups II, III, and IV that were handled with LVC solution, LVC NFs and CERs -NFs revealed no alternations in epidermal and dermal cells related to treated skin sections from the control group (group I) (Figure 8). These outcomes provided that NFs had adequate acceptability toward the skin.

## 3. Materials and Methods

### 3.1. Materials

Levocetrizine dihydrochloride (LVC) was provided by Global-Napi Pharmaceutical Co. (Cairo, Egypt). Fluorescein diacetate (FDA), phospholipid (PC), and polyurethane (PU) were purchased from Sigma-Aldrich (St. Louis, MO, USA). Pluronic F127 was gifted from BASF Co. (New Jersy, NY, USA). Ceramide IIIB and VI were provided by Evonic Co. (GmbH, Germany). Acetone, dimethylformamide (DMF), chloroform, and methanol were obtained from Merck (Darmstadt, Germany). Hydroxypropyl methylcellulose HPMC K4M was purchased from Colorcon (Kent, UK).

### 3.2. Methods

#### 3.2.1. Preparation of LVC-CERs

CERs were formulated employing two types of ceramides, namely, ceramide IIIB and ceramide VI, at 25 mg by thin-film hydration (Table 4). Firstly, PC in different amounts (50, 75, and 100 mg) and ceramides with Pluronic F127 (25 mg) were weighed and dispersed in 10 mL methanol: chloroform (1:2, *v*/*v*). By maintaining pressure in a vacuum for 30 min, the organic phase was gradually vaporized at 60 °C by employing a rotatory evaporator (Heidolph, Burladingen, Germany) at 90 rpm. A film was thereby produced. This was then hydrated utilizing 10 mL distilled water containing 75 mg LVC at 60 °C, which is beyond the transition temperature [41]. For the total hydration of the film, beads were utilized for 45 min and CERs were left in fridge overnight [27].

#### 3.2.2. Characterization of LVC-CERs

##### EE%

The CERs dispersion for the formulae was separated at 20,000 rpm for 60 min at 4 °C utilizing a centrifuge (Sigma 3K 30, Osterode am Harz, Germany). Then, the pellet was solubilized in methanol and analyzed at λ_max_ 231 nm using a spectrophotometer (Shimadzu UV1650 Spectrophotometer; Shimadzu Corp., Kyoto, Japan). EE% was estimated by employing the direct method [42,43].

##### PS and PDI

The PS and PDI were evaluated for the fabricated CERs and Zeta potential (ZP) was evaluated for the optimum CERs using a Malvern Zetasizer (Malvern Instruments Ltd., Malvern, UK) [44,45].

#### 3.2.3. Experimental Design

Using Design expert^®^ version 13, a 2^1^.3^1^ factorial design was structured to evaluate the impact of several factors in formulating CERs. The design involved the performance of 6 runs. Two factors were studied: ceramide type (X_1_) and PC amount (X_2_), which were chosen as independent variables; and EE% (Y_1_), PS (Y_2_), and PDI (Y_3_), which were selected as dependent variables (Table 4). Next, the optimized CERs formula selection was based on the desirability function, which enabled the analysis of all responses. It was decided to select a CERs formula with the least PDI and the highest EE% and PS. The optimized formula was subjected to subsequent characterization.

#### 3.2.4. Effect of Storage

The optimized CERs formula was kept in a refrigerator for 90 days, and its stability was then examined by comparing the PS, PDI, EE% and ZP values of the stored formula with those of the freshly assembled one. In addition, CERs were visually examined for any aggregation [44].

#### 3.2.5. Preparation of the Prepared NFs

Coaxial spinneret (NANON–O1A electrospinner MECC Co., Kokura, Japan), composed of 2 needles put together coaxially, was utilized [46]. Two syringe pumps were employed to make the core and shell mixtures. The flow rate was 0.5 mL/h for the core and 1 mL/h for the shell. A high-voltage supply was employed to create voltages of up to 55 kV, and a collector was employed as a receiving plate to gather the NFs. The space between the tip of the needle and the collecting plate was 12 cm. The core solution comprised 1% (*w*/*v*) HPMC K4M, and the shell solution consisted of PU 8% in DMF/acetone in a 7:3 ratio. All electrospinning procedures were carried out at 25 °C and humidity of around 50% [47].

#### 3.2.6. In Vitro Release

Release from LVC-NFs and CERs-NFs (for comparison with that of LVC solution) were obtained using a shaking water bath (JEIO Tech SI-300 Lab Companion (Kyonggi-Do, Korea) at 37 °C in which flasks loaded with phosphate buffer pH 5.5 acted as the receptor medium at 50 rpm. Briefly, samples of about 2 × 2 cm^2^ from NFs mats were cut into transverse sections that contained 7.5 mg LVC, which were then placed into 50 mL beakers, including 50 mL of the receptor media. LVC release from the NFs was assessed over a 12 h period. Typically, a 1 mL sample of the medium was withdrawn for up to 12 h and substituted by fresh media [48]. The in vitro release of LVC from LVC solution was conducted in parallel, where a dialysis bag comprising 1 mL (7.5 mg of drug) was placed in a beaker containing 50 mL of PBS. The procedures were conducted similarly to the in vitro release study involving NFs.

#### 3.2.7. Morphological Assessment of NFs

##### SEM

The morphology and structural properties of CERs after lyophilization and the NFs were inspected using SEM (JSM-6301 F, JEOL Inc., Peabody, MA, USA). Samples were mixed with 20 nm gold to prevent charging. The distribution of NFs was evaluated by analysis of SEM micrographs [36].

##### AFM

The micrographs of plain fibers (without CERs or LVC), LVC-NFs and CERs-NFs were treated utilizing Gwyddion^®^ to examine their roughness performance. For each formulation, 3D images were handled. The micrographs were eliminated to prevent unnecessary limit values. Consequently, the dependence of the roughness parameters on the deviation in composition was anticipated in nanometers [36].

##### TEM

Morphology of the optimized CERs, LVC-NFs, and CERs-NFs was analyzed employing TEM (Joel JEM 1230, Tokyo, Japan). The samples were positioned on a grid, stained, and then photographed [44,49].

##### CLSM

CLSM (Nikon, Tokyo, Japan) was utilized to explore the allocation of CERs inside NFs. At first, CERs and NFs were fabricated using the amounts and procedures previously stated, except that 1% of the FDA was added to the formulation to replace the amount of LVC in the formulations [18,50].

#### 3.2.8. In Vivo Studies

##### Animals

Approval of the investigation was acquired from the Animal Research Committee of Misr University for Science and Technology (no IP05b 6 June 2022). Eighty-four male Wister rats weighing 150–200 g, with age of 7 weeks, were used. The rats were housed in cages at 22 °C and humidity of 55%. A total of 72 rats were employed in the dermatokinetic study, and 12 rats were utilized in the histopathological examination. For the application of LVC solution, bottle caps were employed; these served as drug pools with an area of 4.91 cm^2^. The caps were positioned on the dorsal skin, which had been shaved before application. Moreover, the samples were placed non-occlusively [25]. For NFs application samples, about 2 × 2 cm^2^ from NFs mats were cut into transverse sections and fixed with the aid of adhesive tape.

##### In Vivo Dermatokinetic Assessment

Animals were classified into three groups of twenty-one animals each. Group I was administered an LVC solution, while groups II and III received LVC-NFs and CERs-NFs topically, respectively. One milliliter of LVC solution equivalent to 7.5 mg LVC was administered to dorsal rat skin [12]. For NFs application samples, about 2 × 2 cm^2^ from NFs mats were cut into transverse sections that contained 7.5 mg LVC from LVC-NFs and CERs-NFs. After therapy, three animals from each group were sacrificed at various time intervals (1, 2, 4, 6, 8, 10, and 12 h). The excised skin was cut into pieces and sonicated in 5 mL methanol for 30 min. The extract was then filtered through a 0.45 μ filter, and HPLC (Shimadzu, Kyoto, Japan) was used to assess the concentration of LVC [51]. Dermatokinetic data were evaluated, and the different dermatokinetic parameters such as T_max_, C_max_, and AUC_0–12_ were estimated utilizing Kinetica^®^ (Thermo Fisher Scientific Inc., Waltham, MA, USA) [52]. C_max_. The values of AUC_0–12_ for the previously treated groups were statistically analyzed by employing a one-way ANOVA statistical test. Further, a signed-rank test was utilized to assess the medians of T_max_ for the treated groups using SPSS^®^ software 22.0. For multiple comparisons, post hoc testing was conducted.

##### Histopathologic Evaluation

In this study, twelve rats were partitioned into four groups of three, and the treatment duration was 1 day. Group I was set as the control, and groups II, III, and IV were treated with LVC solution, LVC-NFs, and optimized CERs-NFs, respectively. Skin autopsies were fixed in 10% formalin. Specimens were affixed and then sectioned by microtome (Leica Biosystems, Wetzlar, Germany). The sections were histopathologically analyzed utilizing light microscopy (ZEISS, Oberkochen, Germany) [25].

## 4. Conclusions

In this study, levocetirizine dihydrochloride (LVC) was loaded into cerosomes (CERs) that were successfully incorporated into electrospun nanofibers for topical treatment of atopic dermatitis. Firstly, LVC was incorporated into CERs using thin-film hydration via a 2^1^.3^1^ factorial design. The optimized CERs showed good entrapment efficiency and optimum particle size for topical administration. The optimized CERs showed good stability during the storage period. Then, the optimized CERs were loaded using the coaxial electrospinning technique into polyurethane (PU) nanofibers (NFs). The NFs were assessed regarding in vitro release surface morphology using scanning electron microscopy, atomic force microscopy, transmission electron microscopy, and confocal laser microscopy. Further, the amount of LVC deposited in the skin was revealed using a dermatokinetic study and compared to that of LVC-NFs. Finally, the safety profile of the optimized CERs-NFs was determined after topical application on the rat’s skin surface. This research successfully concluded that LVC loaded in CERs-NFs sustained the antihistaminic effect of LVC.

## Figures and Tables

**Table 1:** Output data for the 2^1^.3^1^ factorial analysis.

Responses	EE%	PS (nm)	PDI
Adjusted *R*^2^	0.92	0.86	0.80
Predicted *R*^2^	0.83	0.71	0.62
Adequate precision	13.88	10.59	8.73
Predicted value of CER6	65.62	689	0.49
Observed value of CER6	65.03	680	0.51

**Table 2:** Experimental runs of the 2^1^.3^1^ factorial design of LVC-CERs.

	Ceramide Type	PC Amount (mg)	EE%	PS (nm)	PDI	Drug Loading (%)
CER1	IIIB	50	43.15 ± 3.23	531.66 ± 19.39	0.229 ± 0.0005	14.38 ± 0.01
CER2	IIIB	75	51.66 ± 2.49	540.66 ± 7.13	0.366 ± 0.067	12.29 ± 0.23
CER3	IIIB	100	59.05 ± 2.32	648.33 ± 7.76	0.675 ± 0.025	11.81 ± 0.67
CER4	VI	50	46.60 ± 1.67	589.85 ± 0.15	0.548 ± 0.011	15.53 ± 0.98
CER5	VI	75	53.44 ± 1.01	615.00 ± 5.00	0.460 ± 0.017	13.36 ± 0.78
CER6	VI	100	65.03 ± 1.07	680.00 ± 39.50	0.600 ± 0.100	13.00 ± 0.02

**Table 3:** Surface roughness parameters of plain fibers, LVC-NFs, and CERs-NFs

Samples	R_a_	R_q_	R_t_	R_p_	R_tm_
Fiber without LVC or CERs	49.57 ± 6.40	64.62 ± 11.50	532.59 ± 147.50	305.64 ± 71.80	410.89 ± 106.90
LVC-NFs	46.27 ± 5.40	60.32 ± 10.10	511.39 ± 140.40	285.64 ± 65.20	403.27 ± 104.40
CERs-NFs	42.06 ± 4.00	54.37 ± 8.10	473.42 ± 127.80	168.95 ± 36.30	385.42 ± 98.40

Abbreviations: R_a_—root mean square roughness; R_q_—the maximum height of the roughness; R_t_—maximum roughness peak height R_p_, and an average maximum height of the roughness R_tm_; LVC—levocetirizine dihydrochloride; CERs—cerosomes; NFs—nanofibers.

**Table 4:** The 2^1^.3^1^ factorial design for optimization of LVC -CERs.

Factors	Levels
X_1_: Ceramide type	IIIB VI
X_2_: PC amount (mg)	50 75 100
Responses	**Constraints**
Y_1_: EE (%)	Maximize
Y_2_: PS (nm)	Maximize
Y_3_: PDI	Minimize

## Data Availability

The original contributions presented in the study are included in the article, further inquiries can be directed to the corresponding author.
